# Food Accessibility in the Suburbs of the Metropolitan City of Antwerp (Belgium): A Factor of Concern in Local Public Health and Active and Healthy Aging

**DOI:** 10.3390/ijerph192315754

**Published:** 2022-11-26

**Authors:** Bart Geurden, Jeroen Cant, Joris Beckers

**Affiliations:** 1 Centre for Research and Innovation of Care (CRIC), Faculty of Medicine and Health Sciences, University of Antwerp, 2000 Antwerp, Belgium; 2 Center for Gastrology and Primary Food Care, 3000 Leuven, Belgium; 3 Reference Site Three Rivers FoodDelta, EIP on AHA Reference Site Collaborative Network (RSCN), 1040 Brussels, Belgium; 4 Research Group for Urban Development, Faculty of Design Sciences, University of Antwerp, 2000 Antwerp, Belgium; 5 Department of Transport and Regional Economics, Faculty of Business and Economics, University of Antwerp, 2000 Antwerp, Belgium

**Keywords:** food, food accessibility, food insecurity, aging, healthy aging, mobility, malnutrition, e-commerce

## Abstract

Population aging and declining birth rates are key demographic trends of the 21st century. While the overall life expectancy and healthy life years increase, the quality of life and functional capacity worsens due to non-communicable diseases strongly related to aging. Therefore, aging citizens are often vulnerable to food insecurity. The aim of this paper is to provide insights into the physical accessibility of fresh food and possible alternatives within the setting of an aging society in Antwerp (Belgium), a metropolitan city at the heart of the EU Reference Site ‘Three Rivers Food Delta’. We demonstrate that a large number of the Antwerp suburban areas in which 15 to 25% of current inhabitants are already over 65 years old are confronted with problematic physical accessibility of food due to long walking distances to the nearest food shop. E-commerce has the potential to provide better access to fresh food. This is especially relevant for people with specific needs, such as health-related diets, dysphagia, and/or limited mobility. However, e-commerce introduces new inequalities, as those who would benefit the most from digital accessibility currently use it least. Hence, the organization of fresh food access requires a more thoughtful organization of the ‘last mile’ and possible alternatives to home delivery. This makes food accessibility an urgent factor of concern in public health and healthy aging in the Antwerp suburban areas.

## 1. Introduction

### 1.1. Aging and Limited Mobility

Europeans are living longer than ever before and the age profile of society is rapidly developing. In 2021, more than one-fifth (20.8%) of the EU population was aged 65 and over, and the share of people aged 80 years or above is projected to have a two-and-a-half-fold increase between 2021 and 2100, from 6.0% to 14.6% [1]. Consistently low birth rates and higher life expectancy are transforming the shape of the EU’s age pyramid. The most important change will be the steady transition towards a much older population structure, a development that is already apparent in several EU Member States. This has forced communities and people themselves to reshape aging concepts and approaches. In this context, the World Health Organization (WHO) developed a consensus statement [2] promoting a new approach to aging and resilience. Maintaining intrinsic capacity and self-care in a lifelong approach is now becoming central to care concepts for aging citizens around the world. The engagement needs of older people with a focus on functionality rather than disease management as a primary objective are considered an overarching concept, also embracing adherence, compliance, empowerment, health literacy, shared decision making, and activation [3]. However, it is still the case today that many elderly EU citizens have a worsening quality of life and intrinsic functional capacity due to non-communicable diseases that are strongly linked to the aging process [4]. The Longitudinal Aging Study Amsterdam (LASA) confirms that the prevalence of functional disability is increasing and has become a main indicator of health at older ages [5]. Meanwhile, mobility is the most studied and most relevant physical ability affecting intrinsic capacity, self-care, and quality of life with strong prognostic value for disability and healthy aging [6].

In old age, malnutrition is considered a major risk factor for frailty, sarcopenia, impairment, and disability and is becoming increasingly prevalent among EU older citizens [7,8,9,10]. The city of Antwerp and the surrounding area, one of the most densely populated regions in the Three River FoodDelta (3RFD), is no exception. One in two elderly people living independently at home in Antwerp and the surrounding area is at serious risk of malnutrition at the time of admission to hospital for elective surgery [11]. About one in three patients in Antwerp who receive regular and long-term professional nursing care at home are at serious risk of malnutrition [12]. This means that the food intake of elderly people living at home in this environment is often problematic. Additional risk factors that mutually influence inadequate food intake and malnutrition include reduced sensations of taste and smell [13], poor dentition [14], polypharmacy [15], depression, and social isolation [16]. Older adults experiencing financial concerns such as poverty or low income may not be able to buy a sufficient amount and quality of food. The financial aspects of access to healthy food have become even more prominent in light of the current war in Ukraine and the successive financial crises in Europe, especially in countries of lower economic status. In the context of public health and the new WHO approach to aging and resilience [2], early identification and management of food insecurity are essential. For this purpose, we describe in this review, the increasingly difficult physical accessibility of fresh food for the aging population in the Antwerp region. We also discuss the electronic accessibility of fresh food as an alternative to physical accessibility.

### 1.2. Antwerp and the 3RFD

**Figure 1:**
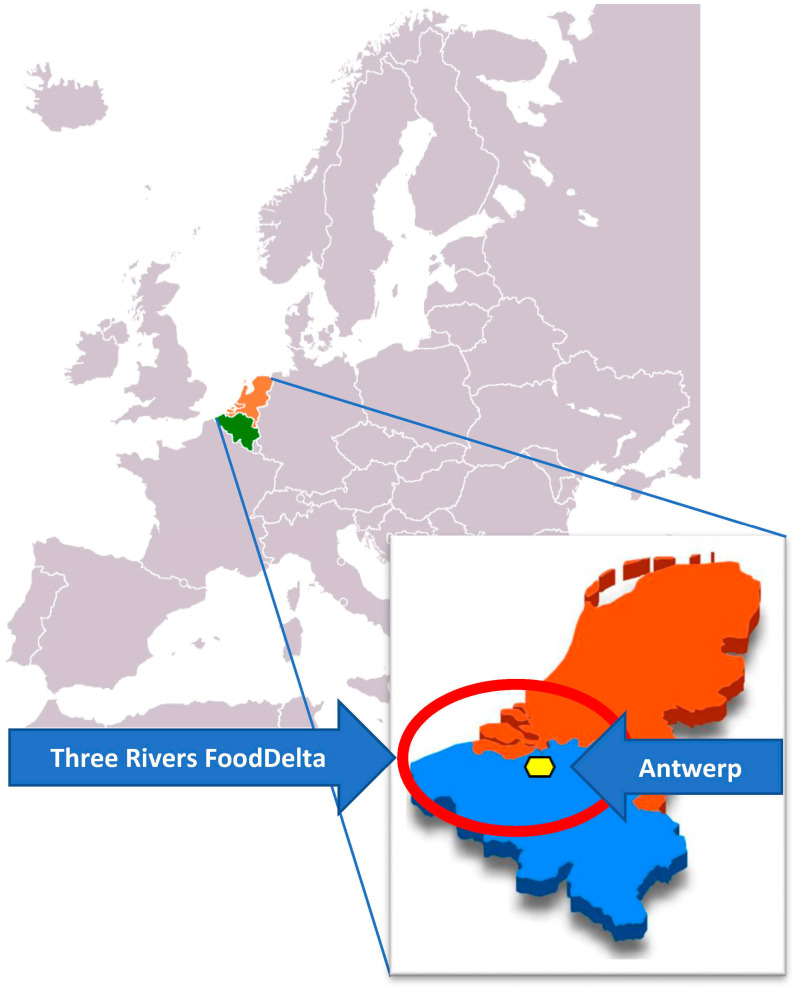
Location of Antwerp in the Three Rivers FoodDelta. Reprinted with the permission of: Center for Gastrology and Primary Food Care, 3000 Leuven, Belgium. 2022, Bart Geurden.

The “Three River FoodDelta” (3RFD) spans the Rhine–Meuse–Scheldt delta region in The Netherlands, extended with three neighboring provinces in Flanders (Belgium) (Figure 1). On 1 January 2022, this transnational area counted 11,343,549 inhabitants (4,610,275 inhabitants on the Belgian side, and 6,733,274 inhabitants on the Dutch side of the border) of which about 22% are aged 65 and over. Rotterdam and Antwerp are the two largest metropolitan cities in 3RFD, each with more than 500,000 inhabitants. In this paper, we will refer to the city of Antwerp and its suburbs.

3RFD is an EU Reference Site and is a member of the Reference Sites Collaborative Netwerk (RSCN). As such, it has become an inspirational ecosystem that brings together regional government, health and care providers, academia, industry, and civil society to drive patient-centered structural changes far beyond the scope any one organization could achieve on its own. 3RFD focuses on gastrological solutions for people with a problematic food intake due to aging or due to illness and/or treatment. This culi-clinical approach aims to optimize food-related quality of life, prevent malnutrition, and facilitate lifelong active and healthy aging.

## 2. Objectives

This paper describes the physical and online accessibility of food in general and for the elderly living in the suburbs of the metropolitan city of Antwerp (Belgium). It combines research results from different lines of research at the University of Antwerp to identify and discuss current gaps in food accessibility in this particular living environment. It also makes recommendations to improve local food accessibility and to conduct further research into the implementation of measures to achieve a smart, healthy, and age-friendly environment.

## 3. Physical Accessibility of Food

Food environments describe the context in which consumers engage with the food system. They are complex with substantial local variations, based on different factors, including geographical, transport, sociocultural, and economic factors [17]. These food environments are likely to have an important impact on the quality of meals and food intake [18]. Well-known and documented accessibility failures of local food environments include food swamps and food deserts. In food swamps, unhealthy food options, typically energy-dense nutrient foods, are overabundant as compared to healthy foods [19]. Food deserts, on the other hand, are areas, usually presumed to be deprived urban neighborhoods, where residents have insufficient access to a mix of foods necessary for a healthy diet [20,21]. In light of this discussion paper, studies of food deserts, i.e., a lack of access to healthy food, are particularly poignant.

Food deserts are a systemic issue in the US as access to healthy food is strongly determined by income and race, while evidence of their existence in other countries is sparse [20]. The scientific literature, however, starts from a strict interpretation of food deserts, focusing solely on deprived neighborhoods. It is assumed the residents of such areas are less mobile and therefore more dependent on their local food environment. It is true that the socioeconomically deprived are less mobile, e.g., due to the cost of purchasing, owning, or operating a car [21,22]. Even with access to a car, they travel less frequently and over shorter distances [23,24]. Moreover, when they do own a car, it is often an older and fuel-inefficient one [22,23], making them more vulnerable to increased fuel prices [25]. The current era in which fossil fuels have become an unreliable and expensive commodity highlights this issue [26]. Moreover, older and fuel-inefficient cars are affected by restrictive automobility measures such as ‘low-emission zones’ (LEZs) [27]. Such a LEZ was introduced in Antwerp on February 25th in 2017 with an application of the Flemish Decrees on low-emission zones (2015 and 2016). For all these reasons, the socioeconomically deprived are much more dependent on their local food environments as compared to the general population.

Many other groups, however, are also confronted by mobility reductions, the elderly being a prime example. They, particularly those aged 75 and over, tend to drive less than their younger peers [28]. This is mostly due to deteriorating physical and mental health, and cognitive ability. Efficient and accessible public transport could be a crucial coping strategy for driving cessation for the elderly [29]. However, public transport in practice is often cumbersome, especially for the elderly, and is regarded as a poor substitute for automobility [30,31]. Relying on others for trips to a food store is another coping strategy. Again, however, the elderly prefers not to, as they fear being a burden [30,32]. Finally, walking abilities significantly decrease with age through increasing gait disorders, especially in frail populations [33]. The COVID-19 pandemic further worsened existing vulnerabilities and inequalities [34]. The elderly also certainly seems to have suffered from decreased mobility during the pandemic as (i) shop closures limited the number of touchpoints; (ii) fear of contamination kept them home; and (iii) restrictions in public transport services limited modal choice [34].

It therefore needs to be concluded that aged citizens are likewise highly dependent on their local food environments. As argued by Shaw [35], a broader definition of food deserts, taking into account all forms of immobility rather than just socioeconomic deprivation, would often be more appropriate to study food accessibility. Such a reorientation seems appropriate, especially in Western Europe, and especially in the 3RFD, where society is highly egalitarian but also rapidly aging.

Our research on the accessibility of food retailing in Flanders [36] confirms the observations of Beaulac et al. [20], i.e., very few food deserts emerge when using the traditional definition of food deserts with a focus on socioeconomic deprivation. Poverty is mostly contained in the major urban areas where a dense network of supermarkets and traditional independent food stores still exists. Similarly, in the centers of most small cities and villages, food accessibility is high and retailers are usually available within walking distance. The execution of most daily activities, including shopping, will therefore still take place in the known and trusted own municipality [37].

**Figure 2:**
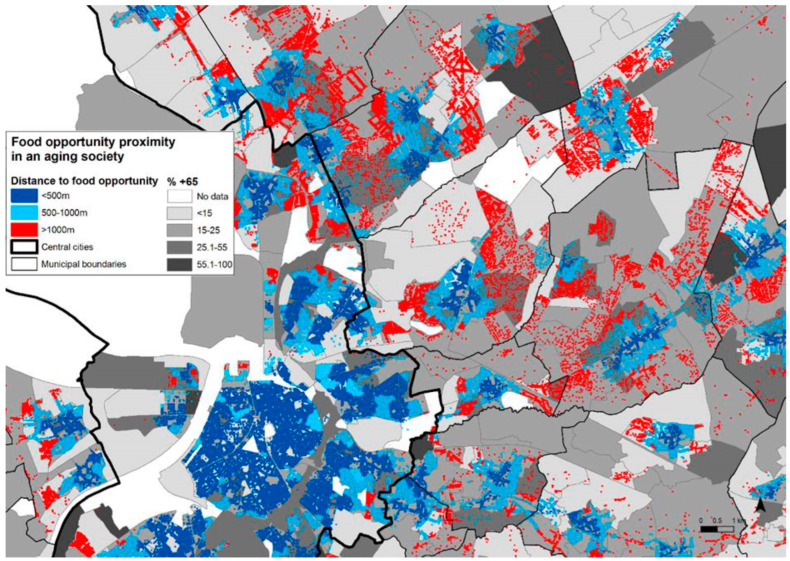
Walking distances to food opportunities and % residents + 65 in Antwerp and its north-eastern suburbs. Reprinted with the permission of [36]. Jeroen Cant, 2022.

However, Flanders has been confronted with significant and unbridled urban sprawl [38]. This sprawl spread particularly rapidly between the 1950s and 1980s and continues to this day. It took the form of monofunctional, low-density neighborhoods. While the low densities in themselves would probably have discouraged a healthy mix of residential and retail functions, strict zoning and building regulations ensured that this actually happened [39]. Currently, the travel distance to the nearest supermarket or other food vendors in many of the Antwerp suburbs is high, and much more than a comfortable walk (See Figure 2). This was never seen as a problem before, as the suburbs were typically populated by members of the highly mobile middle classes [40]. A short drive to the nearest supermarket seemed normal and it was accepted as part of suburban life. However, the suburbs built in the second half of the 20th century are currently aging rapidly [41]. There is a strong desire among these residents to age at home and to delay moving to a nursing home for as long as possible [42,43,44]. This trend is even strongly promoted by the government [45]. In the Antwerp suburbs, this urbanization and healthcare policy has resulted in neighborhoods with both low accessibility scores and rapidly declining mobility rates.

Figure 2 provides an overview of the city of Antwerp (see the lower, left quadrant in Figure 2) and its northeastern suburbs (the other quadrants in Figure 2). Antwerp is the largest city in Flanders and an important destination for commuters [46]. The Antwerp suburbs were therefore the first in the region to be confronted with substantial monofunctional, low-density urban sprawl. Light blue dots in Figure 2 represent areas within 500–1000 m walking distance of a place where grocery needs can be fulfilled. This might be a supermarket or a local clustering of traditional food stores. The red-colored dots and areas are more than 1000 m walking distance away from food shops. Opportunities within a 500 m distance have been shown to have a positive influence on diet patterns [47,48]. Longer distances might still be acceptable for relatively fit people, though, beyond 1000 m, the walkability of food stores becomes spurious. Addresses in the center of Antwerp city itself (see left, lower quadrant in Figure 2) are generally colored dark blue, which means that the walking distance to the nearest food store is less than 500 m. This is also true for the historical village cores of the Antwerp suburbs. However, most of the urban sprawl is colored red and therefore not considered to be within a walkable distance.

The map with red and blue-colored dots and areas is then superimposed on a map showing the distribution of the percentage of the population aged 65 and over (grey shades in Figure 2). A significant number of red dots and areas can be found in places where at least 15%, and up to 25%, of residents are 65 years of age and older [49]. It is expected that the current inhabitants of these Antwerp suburban areas will age considerably further in the coming decades.

Without an adequate plan of action, this trend of aging in an inappropriate environment will have an increasingly negative impact on the accessibility of healthy food and therefore the quality of meals and daily food intake. This, in turn, will be a factor that reduces quality of life in general and puts a strain on active and healthy aging.

It will, however, be difficult to introduce brick-and-mortar retail to the neighborhoods discussed: low densities mean the local markets are too small to be profitable, there is a lack of suitable land and real estate, and local zoning laws could preclude commercial activities. Innovative distribution methods need to be explored. These should go beyond the basic provision of healthy food items. Indeed, retail also provides important social functions and interactions at shops, which are important for the elderly in loneliness alleviation [50,51]. Distribution innovations should thus fulfill the full retailing experience.

## 4. Online Accessibility of Food

E-commerce is expected to increase access to goods and services [52,53] and reduce at least part of the inequalities mentioned above. Indeed, by moving transport (almost) entirely to the supply side, possible mobility issues can be overcome. However, the availability of home delivery services and the propensity to shop online are putting new constraints on the relationship between the supply and demand of goods and services. A growing body of research shows that young highly educated professionals, in particular, i.e., the most mobile groups suffering the least from inaccessibility, represent the bulk of online demand [54,55,56]. This is also the case for online grocery demand in particular [57,58]. Moreover, even the staunchest online shoppers still regularly visit physical grocery stores [59]. Possible negative repercussions on the physical retail landscape could then, theoretically, even further decrease food store accessibility for the most vulnerable groups. Nevertheless, the use of the Internet as a new additional sales channel has already proven beneficial for the socially excluded and people with reduced mobility [60,61], and there is proof of rural and semi-rural areas at the city fringe with high demand for e-commerce and other Internet services in the UK [58]. Whether e-commerce will eliminate food deserts remains to be seen, as the relationship between physical retail supply and online demand is complex, with evidence supporting both the innovation diffusion hypothesis, meaning greater uptake in urban areas, and the efficiency hypothesis meaning greater uptake in areas with a low physical store supply [62,63].

**Figure 3:**
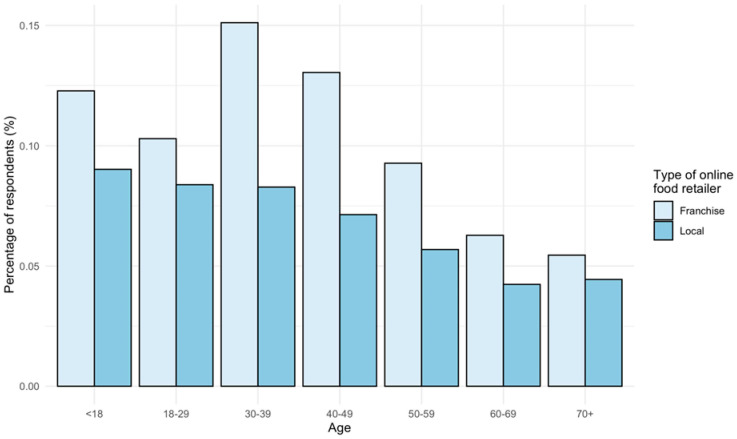
Percentage of respondents that increased their online food shopping frequency. Reprinted with the permission of [64]. Joris Beckers, 2021.

The COVID-19 pandemic proved to be a catalyst for e-commerce engagement, increasing the frequency of online shopping across different product categories and by people from diverse socio-demographic backgrounds [64,65,66]. This observation holds up specifically for groceries [57,67]. The elderly too found their way online, yet age remained a significant relevant inhibiting factor [57,64]. Figure 3 demonstrates the age effect on the uptake of online food purchasing [64]. The uptake was measured in 2020 compared to the year before. Franchise retailers are presumed to be e-grocers; local food retailers are restaurants. Moreover, older age groups are significantly more likely to go back to physical grocery shopping after the pandemic [34]. On the other hand, learned habits combined with a loss of mobility is an important condition for future online grocery shopping [68].

Home deliveries represent a new dimension in the delivery of goods, causing significant cost increases due to vastly increased fragmentation. Logistics service providers are challenged to cope with high competition, a consumer-driven economy, failed delivery issues, reverse logistics, and environmental measures taken by policymakers, which are all putting pressure on the bottom line [69]. In many European oligopolistic grocery markets, see, e.g., Hirsch and Koppenberg [70], these costs are often still passed on to consumers. Major retailers in Flanders, for example, still charge substantial delivery costs and prices online are often more expensive than in stores. Moreover, many delivery services do not cover the entire territory and have minimum order sizes. Delivery charges may be dropped for large deliveries, but this would not often apply to the elderly who mostly live in small family units.

The recent case of Gorillas, an on-demand grocery delivery service that seized operations in Antwerp and Brussels after just one year, indicates there is currently still little room for disruption in the Flemish market. On the other hand, non-exclusive grocery retailers such as Amazon might be able to compete on prices through cross-subsidization with other product/service categories [71]. While they may have the financial backing to penetrate a closed market, their presence in the European groceries market is still marginal and completely non-existent in Flanders. Furthermore, even in more mature home delivery markets such as the US, fees and minimum order requirements continue to exist anyways [72], restricting access for those that need it most [73].

Recently, Sanchez-Diaz et al. [74] and Newing et al. [75] studied e-commerce accessibility. The former groups zip codes in western Sweden in clusters of accessibility based on the level of delivery services for prescribed drugs; the availability of food delivery services; and the vicinity and count of delivery services provided by three logistics service providers. The latter study quantifies the provision of healthy grocery products in the UK. Physical retail is included through data of traditional retail outlets, transport infrastructure, and small area socio-economic indicators. E-commerce accessibility is measured through an index that combines the count of retailers offering home delivery and the demand for online services. In Sweden and the UK, inequalities are found in home delivery services with the lowest accessibility in more run-down areas. E-commerce did improve overall access to groceries in Britain’s rural areas; although, areas with a low supply of physical retail benefited the least from online services.

One may then conclude that the current system of e-commerce with home delivery is inadequate to address the issues of inaccessibility in immobile groups discussed in the previous section, in particular the elderly. First of all, there seems to be a spatial mismatch, with those needing it most having less access. Empirically, they also use such services substantially less than younger age groups, even in times of crisis, such as during the COVID pandemic. The system is also poorly adjusted to them, making home deliveries particularly expensive or even impossible for the elderly, likely further hampering uptake.

Continuous innovation in last mile delivery schemes, however, has the potential to increase the overall accessibility to e-groceries, complementing the existing availability of services [76]. The price and sustainability of the ‘last mile’, the last part of the supply chain when home delivery actually takes place, have recently been the focus of a significant amount of research. A common denominator of these studies is the identification of delivery alternatives that might postpone the point of fragmentation within the supply chain for as long as possible and prevent failed deliveries/re-delivery. One such alternative, collection-and-delivery points (CDPs), is offering the opportunity to consolidate all parcels destined for a particular neighborhood within one logistics hub [77]. With minimal investments in infrastructure, CDP can lead to significant reductions in costs [69,78,79] and environmental impact [80,81] on the supply side. This provides an opportunity to lower or abolish fees and minimum order sizes for grocery deliveries [73]. In addition to their logistical role, CDPs can also play a social role by acting as a point of contact within the neighborhood [82]. This became especially relevant during the COVID-19 pandemic. Finally, CDP could decrease walking times for grocery pick-ups [82].

CDP can take various forms, from simple automated lockers to using the existing network of small shops. The latter approach even showed promise in the US, though there were some low-density neighborhoods where the system might not be worth it due to limited service levels [73]. A study in Christchurch, New Zealand, shows that using small shops allows for creating a walkable and bikeable CDP system [83]. In Flanders, and indeed most of Europe, there, likewise, still exists such a dense network of convenience and other traditional stores [36].

Using small local shops has many other benefits from the point of view of the elderly. For one, it optimizes the social role of the CDP. Encounters do not depend on hasty chance encounters but rather take place in a more traditional shopping atmosphere. Moreover, shop assistants can entice people to buy online or provide assistance to increase usage. Web design (or the lack thereof) has a strong impact on the perceived level of trust [84] and may prevent more cautious elderly to shop online. Help in this initial step would aid in overcoming this fear. This is certainly important when rolling out a CDP program, since initially acquiring online shopping habits in the elderly is an important predictor of future use [68].

There are several pitfalls to the CDP system though. For example, current CDP networks have grown largely organically, and lack the professionalization needed to fully exploit the potential economic, environmental, and social benefits [85]. For grocery deliveries, in particular, CDP is still in its infancy and the standard delivery model still organizes e-groceries deliveries through store-based fulfillment, i.e., in-store pick-up [86]. This has no beneficial impact on accessibility. There is an obvious need for a better-planned CDP network and supermarkets should be encouraged to adopt them.

The new home delivery offer Is also generating new inequalities. The impact of density is severe and costs vary by a factor of 10 between the city centers of Brussels and Antwerp on the one hand, and most of the rural regions in the southern part of Belgium on the other [69]. The contrast in delivery costs has a significant impact on the supply chain design. From a cost-optimizing perspective, CDP densities should then decrease in the suburbs and rural areas, reintroducing a need for automobility. Alternatively, higher fees could be charged to the inhabitants of lower-density neighborhoods. Either would be detrimental to the accessibility benefits of CDP. Therefore, a socioeconomically optimized CDP requires taking into account consumer characteristics [73,83]. This may increase the price at the supply side compared to an idealized scenario, but can improve customer uptake while there would still be very significant cost savings as compared to home delivery [73].

## 5. Conclusions

A large number of the Antwerp suburban areas at the heart of 3RFD are confronted by long walking distances to the nearest food shop. Given that 15 to 25% of current residents are already 65 years of age and over, physical access to food in these areas is becoming increasingly problematic.

E-commerce has the potential to provide better access to fresh food. This is relevant for the elderly and those with special needs, such as health-related diets, dysphagia, or limited mobility. However, e-commerce introduces a new inequality as those who would benefit the most from it currently benefit the least. Therefore, the following measures are recommended to policymakers and all relevant stakeholders in the 3RFD region, and in the Antwerp region in particular. To better exploit the benefits of e-commerce in the context of accessibility, integrated urban planning of retail, mobility, and freight is needed. The reorganization of better access to fresh food also requires a more thoughtful and adequate organization of the ‘last mile’ and the development of possible alternatives to home delivery. To realize smart, healthy, and more age-friendly environments, new interdisciplinary research approaches need to be established that are better aligned with the multidimensional needs of the local aging population. Meanwhile, food accessibility continues to be an urgent and increasing concern in public health and healthy aging in the Antwerp suburbs.

Although in this manuscript we focus on 3RFD and the Antwerp region as an example, the results and recommendations can be translated to many other regions and countries in Europe and beyond. The problems that cause inaccessibility in the research region, namely, low density, rapid aging, and the suburbanization of the elderly, are indeed spread internationally. The demand for attention on the part of government and local policy for these specific problems is urgent. The proposed digital interventions can be applied in different places in the same way in order to create smart, healthy, and age-friendly environments.

## Data Availability

Not applicable.
